# Integrated Cytotoxicity and Metabolomics Analysis Reveals Cell-Type-Specific Responses to Co-Exposure of T-2 and HT-2 Toxins

**DOI:** 10.3390/toxins17080381

**Published:** 2025-07-30

**Authors:** Weihua He, Zuoyin Zhu, Jingru Xu, Chengbao Huang, Jianhua Wang, Qinggong Wang, Xiaohu Zhai, Junhua Yang

**Affiliations:** 1 Institute of Pet Science and Technology, Jiangsu Agri-Animal Husbandry Vocational College, Taizhou 225300, China; 2008020102@jsahvc.edu.cn (W.H.);; 2 Institute for Agro-Food Standards and Testing Technology, Shanghai Academy of Agricultural Sciences, Shanghai 201403, China; zhuzuoyin123@163.com (Z.Z.);

**Keywords:** T-2 toxin, HT-2 toxin, porcine cells, metabolic pathways, biomarkers, combined toxicity

## Abstract

T-2 toxin and HT-2 toxin are commonly found in agricultural products and animal feed, posing serious effects to both humans and animals. This study employed combination index (CI) modeling and metabolomics to assess the combined cytotoxic effects of T-2 and HT-2 on four porcine cell types: intestinal porcine epithelial cells (IPEC-J2), porcine Leydig cells (PLCs), porcine ear fibroblasts (PEFs), and porcine hepatocytes (PHs). Cell viability assays revealed a dose-dependent reduction in viability across all cell lines, with relative sensitivities in the order: IPEC-J2 > PLCs > PEFs > PHs. Synergistic cytotoxicity was observed at low concentrations, while antagonistic interactions emerged at higher doses. Untargeted metabolomic profiling identified consistent and significant metabolic perturbations in four different porcine cell lines under co-exposure conditions. Notably, combined treatment with T-2 and HT-2 resulted in a uniform downregulation of LysoPC (22:6), LysoPC (20:5), and LysoPC (20:4), implicating disruption of membrane phospholipid integrity. Additionally, glycerophospholipid metabolism was the most significantly affected pathway across all cell lines. Ether lipid metabolism was markedly altered in PLCs and PEFs, whereas PHs displayed a unique metabolic response characterized by dysregulation of tryptophan metabolism. This study identified markers of synergistic toxicity and common alterations in metabolic pathways across four homologous porcine cell types under the combined exposure to T-2 and HT-2 toxins. These findings enhance the current understanding of the molecular mechanisms underlying mycotoxin-induced the synergistic toxicity.

## 1. Introduction

Mycotoxins are secondary metabolites produced by various fungal species such as *Aspergillus*, *Penicillium*, or *Fusarium* [1]. According to the Food and Agriculture Organization (FAO), up to 25% of the world crop production is contaminated with varying degrees by mycotoxins, threatening human health [2]. T-2 toxin (T-2) is the most toxic mycotoxin among type A trichothecenes, and HT-2 toxin (HT-2) is its primary metabolite. Both are mainly produced by *Fusarium* species and are frequently detected in various major cereal-based food products worldwide [3,4]. A survey conducted in the European Union (EU) showed an incidence of contaminated samples of 20% (out of 3490 samples) and 14% (3032 samples) for T-2 and HT-2 toxins, respectively [5]. In cereal crops from Sichuan Province-China, the incidence of HT-2 toxin was 49.7% with an average concentration of 3.7 μg/kg, while the T-2 toxin was present in 11.6% of samples at an average concentration of 0.5 μg/kg [6]. T-2 and HT-2 toxins are commonly detected contaminants in cereal crops and frequently co-occur in the same samples [7,8,9].

Extensive evidence has demonstrated that the T-2 toxin and HT-2 toxin exert a broad spectrum of toxic effects in both humans and animals, inducing the damage of the skin, immune system, liver, reproductive system, and gastrointestinal tract [10,11]. In pigs, the toxic effects of the T-2 toxin are largely due to the combined action of T-2 and HT-2 toxins, which are the sole detectable metabolites produced by intestinal microbiota [12]. Furthermore, the immune system is one of the main toxic targets of the T-2 toxin, and repeated exposure to the T-2 toxin has been shown to lead to immunosuppression and reduced resistance to various infectious diseases in animals [13]. Taroncher et al. [14] reported that the combined exposure of T-2 and HT-2 toxins exhibited antagonistic effects in HepG2 cells. Our previous research showed that mitochondrial damage induced by oxidative stress under T-2 toxin exposure may lead to apoptosis in human liver cells [15]. Moreover, we observed that the co-exposure of T-2 and HT-2 toxins to porcine Leydig cells exhibited synergistic cytotoxicity at low concentrations, whereas antagonistic interactions were detected at higher concentrations [16]. Although the combined toxicity of T-2 and HT-2 toxins has been studied in individual cell lines, their differential effects across multiple porcine cell types have not been systematically investigated.

Pigs are among the most sensitive animals to a wide range of mycotoxins [17,18,19,20]. With the rapid growth of the pig-farming industry, the impact of T-2 toxin- and HT-2 toxin-contaminated feed on China’s livestock production has become increasingly serious [21]. Metabolomics, a powerful systems biology approach, enables the comprehensive profiling of biochemical alterations in response to toxicant exposure [22,23]. For instance, LysoPC (16:0) was identified as a biomarker of kidney injury in mice, with its differential expression influenced by aflatoxin M1 (AFM1) and ochratoxin A (OTA) exposure [24]. Similarly, metabolomics analysis revealed that deoxynivalenol (DON) downregulates unsaturated fatty acid biosynthesis and fatty acid metabolism in porcine intestinal epithelial cells (IPEC-J2) [25].

To investigate the cellular responses to the combined exposure of T-2 and HT-2 toxins, four distinct porcine cell types were selected: IPEC-J2, porcine Leydig cells (PLCs), porcine ear fibroblasts (PEFs), and porcine hepatocytes (PHs). These cell models represent key physiological barriers and target organs involved in mycotoxin metabolism and systemic toxicity. Our study investigated the comparative cytotoxicity of T-2 and HT-2 toxins, individually and in combination, across different doses. The combined toxic effects (synergistic, additive, and antagonistic interactions) were evaluated using combination index (CI) modeling. Additionally, the untargeted metabolomic approach was employed to characterize toxin-induced metabolic perturbations, identify potential signal pathways, and explore biomarkers associated with synergistic effects at low doses. These findings provide valuable insights into mycotoxin-induced metabolic dysregulation and support the identification of potential biomarkers for toxicological risk assessment.

## 2. Results

### 2.1. Toxicity of T-2 and Ht-2 Toxins on Four Porcine Cell Lines

**Figure 1:**
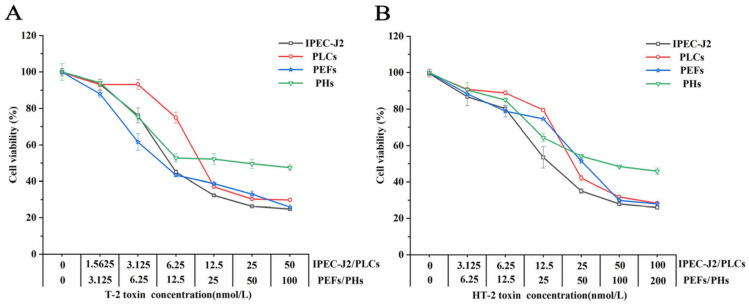
Cytotoxic effects of T-2 (**A**) and HT-2 (**B**) on four different porcine cell lines.

Following 24 h of incubation with a toxin-containing culture medium, cell viability was assessed (Figure 1). The viability of all four porcine cell lines exhibited a dose-dependent decrease with increasing concentrations of the toxins. The half-maximal inhibitory concentration (IC_50_) values for T-2 and HT-2 varied across the different cell lines. Notably, IPEC-J2 was the most sensitive to both toxins, demonstrating the lowest IC_50_ values. In terms of toxicity, T-2 exhibited the greatest effect on IPEC-J2 cells, followed by PLCs, PEFs, and PHs cells. The toxicity profiles of HT-2 and T-2 were similar across the cell lines. As detailed in Table 1, the toxicity of HT-2 in PEFs was found to be 3.26 times greater than that of T-2, while the toxicity in other cell types was approximately twice that of T-2.

### 2.2. Combined Toxicity of T-2 + HT-2 in Four Porcine Cell Lines

**Figure 2:**
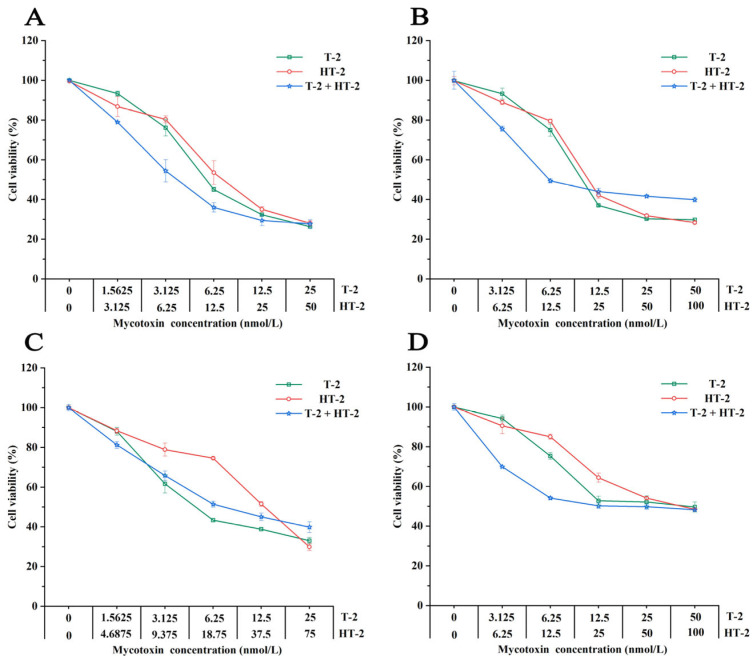
Cytotoxicity of T-2 and HT-2 and their combination (T-2 + HT-2) in four porcine cell types: (**A**) IPEC-J2; (**B**) PLCs; (**C**) PEFs; (**D**) PHs.

The effects of T-2 and HT-2, both individually and in combination, on the cellular viability of four different porcine cell types are presented in Figure 2. Briefly, exposure to both individual and combined mycotoxins resulted in a dose-dependent reduction in cell viability. At lower concentrations, the combination of T-2 and HT-2 exhibited greater cytotoxicity than the single toxins across all four cell types. However, at higher concentrations, the combined treatment was less toxic than the individual toxins in PLCs and PEFs, whereas it remained more toxic in the other two cell types.

### 2.3. Interactive Analysis of T-2 and HT-2 in Four Porcine Cell Lines

**Figure 3:**
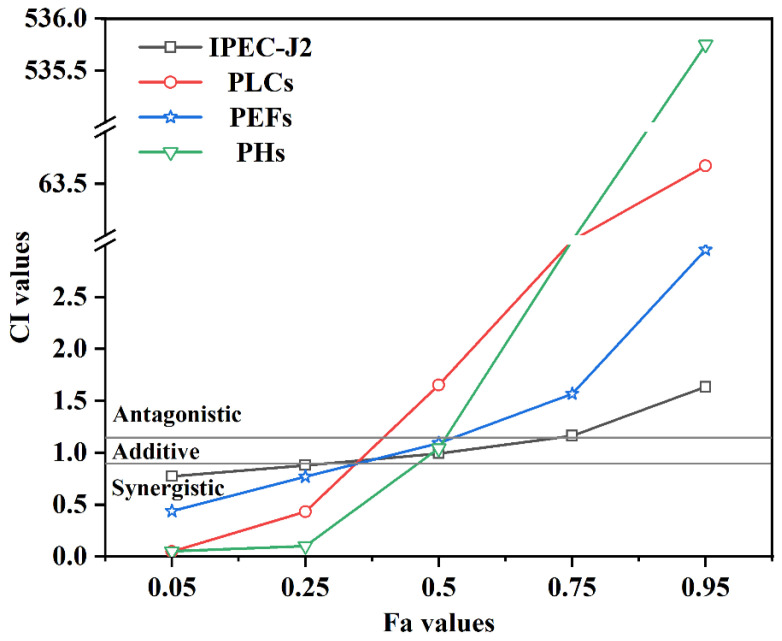
CI-fa curves of four porcine cell lines exposed to a binary mixture of T-2 and HT-2 toxins. CI values between 0.90 and 1.10 indicated additive effects, CI < 0.90 indicated synergistic effects, and CI > 1.10 indicated antagonistic effects. The horizontal gray solid lines represented the lower and upper boundaries of the additivity zone.

The combined cytotoxic effects of T-2 and HT-2 were evaluated using CI-Fa plots and dose reduction index (DRI) values, as presented in Figure 3 and Table 2. The CI-Fa curves (Figure 3) illustrate how the characteristic of the interaction between the two toxins varied with dose. Both toxins exhibited comparable interaction patterns across all four porcine cell lines. A synergistic effect (CI < 0.9) was observed at low cytotoxicity levels (<25%), while an antagonistic interaction (CI > 1) emerged at higher cytotoxicity levels (approximately 75%). To further evaluate the synergism between T-2 and HT-2, DRI values were calculated at 25%, 50%, and 75% cytotoxicity for each cell line (Table 2). At 25% cytotoxicity, all DRI values exceeded 1, indicating a favorable dose reduction in the presence of both toxins. This suggested that the interaction between T-2 and HT-2 was synergistic at low toxicity levels across all tested porcine cell lines.

### 2.4. Evaluation of Experimental Data Quality

**Figure 4:**
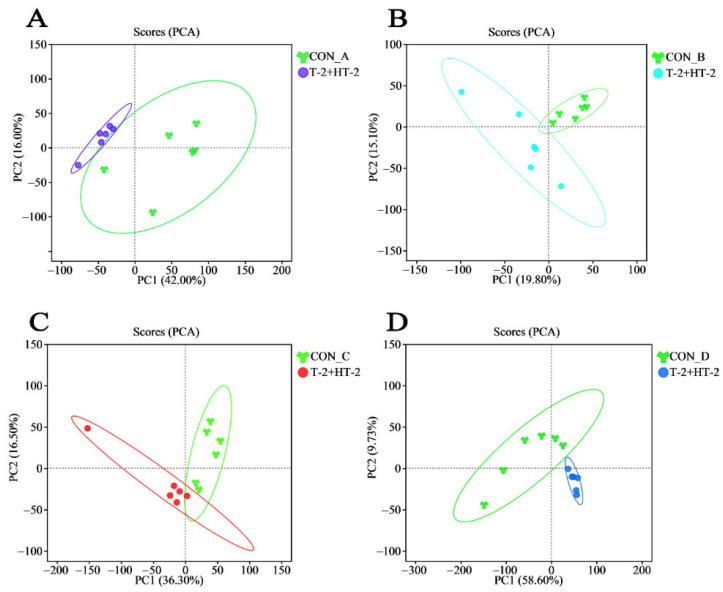
Principal component analysis (PCA) of metabolite profiles in four porcine cell types following the co-exposure to T-2 and HT-2: (**A**) IPEC-J2; (**B**) PLCs; (**C**) PEFs; (**D**) PHs. Y-shaped symbols represent the control group, while circular dots indicate the group treated with the combined T-2 and HT-2.

In the current study, all QC samples were stable and reliable, demonstrating the stability and reproducibility of the analytical instrumentation and confirming the reliability of the data for subsequent analysis (Figure S1). Following co-exposure to T-2 and HT-2, all four porcine cell lines exhibited synergistic effects at the 25% inhibition level. To comprehensively investigate the potential toxicological pathways and identify biomarkers associated with low-dose synergism, an untargeted metabolomic analysis was performed on four porcine cell types using LC-Q-TOF/MS. After data preprocessing, principal component analysis (PCA) models were successfully constructed, revealing distinct metabolic features in each cell line. The PCA score plots clearly demonstrate a distinct separation between the treatment and control groups in four cell lines, indicating that co-exposure led to significant metabolic changes. These significantly altered metabolites may serve as potential biomarkers associated with cellular injury induced by T-2 and HT-2 toxins (Figure 4).

### 2.5. Characteristics of Overall Metabolite Changes

**Figure 5:**
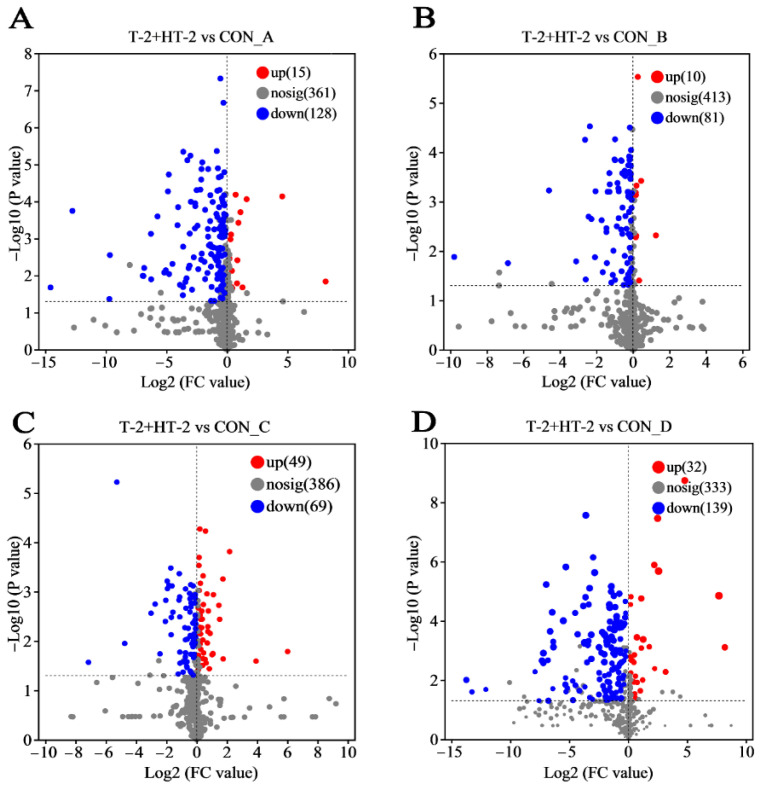
Overview of metabolite alterations in four porcine cell types following co-treatment with T-2 and HT-2 toxins: (**A**) IPEC-J2, (**B**) PLCs, (**C**) PEFs, and (**D**) PHs. Each dot represented an individual metabolite. Red dots indicate significantly upregulated metabolites, blue dots indicate significantly downregulated metabolites, and gray dots represent the metabolites with no significant change.

Multivariate statistical analysis revealed significant alterations in metabolite profiles across the four porcine cell lines following co-exposure to T-2 and HT-2 toxins. Using supervised orthogonal partial least squares discriminant analysis (OPLS-DA), important variables were identified with VIP values > 1 and *p* < 0.05. The changes in differential metabolites between the T-2 + HT-2 treatment group and the control group are depicted in Figure 5. In response to toxin co-exposure, the number of upregulated metabolites in IPEC-J2, PLCs, PEFs, and PHs cells was 15, 10, 49, and 32, respectively. Compared with the control group, the number of downregulated metabolites in the same cell types was 128, 81, 69, and 139, respectively.

### 2.6. Identification and Analysis of Overlapping Metabolites

**Figure 6:**
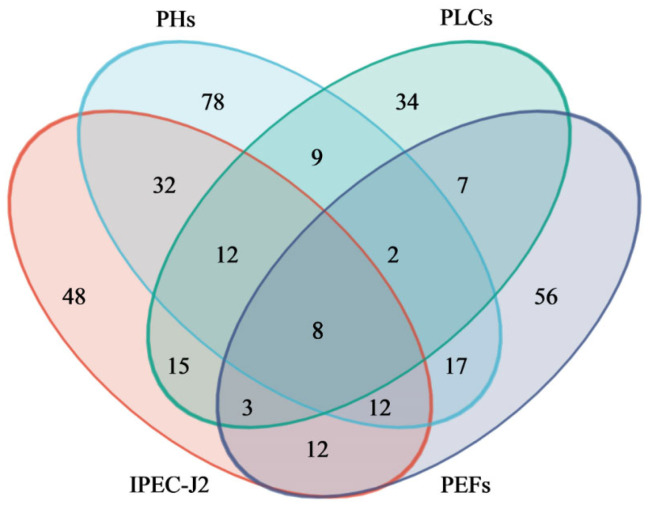
Venn diagram of differentially expressed metabolites in the four cell types following co-treatment with T-2 and HT-2 toxins. Overlapping regions represent the number of metabolites among different cell types, while non-overlapping areas indicate metabolites unique to one single group. The numerical labels correspond to the number of metabolites in each category.

Venn diagrams were employed to visualize the overlap and distribution of endogenous metabolites across the four porcine cell lines (IPEC-J2, PLCs, PEFs, and PHs) following combined exposure to T-2 and HT-2 toxins (Figure 6). Three classes of overlapping metabolites with potential biological significance were identified: lysophosphatidylcholines (LysoPCs), lysophosphatidylethanolamines (LysoPEs), and avenestergenin A1. Five distinct metabolites were classified under LysoPC. Detailed information of these metabolites, including their identifiers, chemical names, molecular formulas, and mass-to-charge (*m*/*z*) ratios, are provided in Table 3.

### 2.7. Analysis of Differential Metabolites

**Figure 7:**
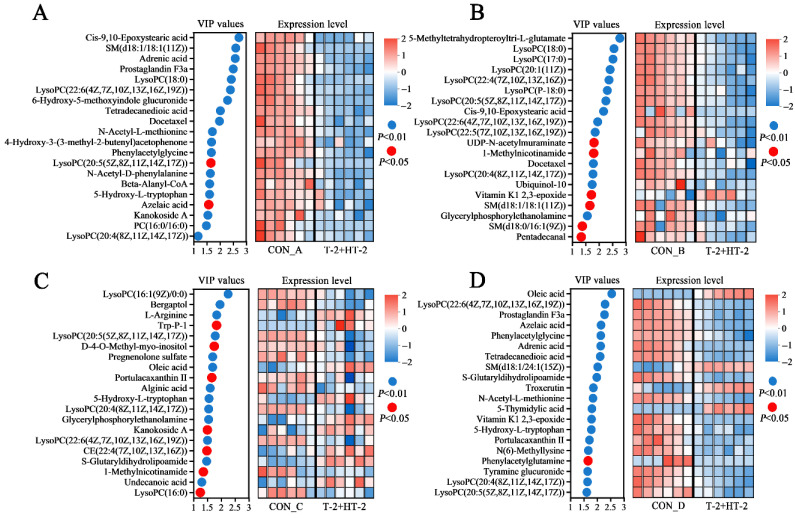
Bubble plots of variable importance in projection (VIP) scores and heatmaps of differential metabolite expression in four porcine cell types following co-treatment with T-2 and HT-2 toxins: (**A**) IPEC-J2, (**B**) PLCs, (**C**) PEFs, and (**D**) PHs. Con_A to Con_D represent the toxin-free culture medium controls corresponding to the co-exposure conditions of T-2 and HT-2 in the four different cell lines. The left panel displays the VIP score bubble plot in each subfigure, where red bubbles denote metabolites with *p* < 0.05, and blue bubbles denote *p* < 0.01. The right panel presents the corresponding heatmap of metabolite expression. Blue gradients indicate downregulation, and red gradients indicate upregulation, with darker colors representing greater magnitudes of change.

To investigate the metabolic disturbances induced by combined exposure of T-2 and HT-2, the top 20 endogenous differential metabolites (ranked by composite scores and annotated with KEGG identifiers) were selected for each of the four porcine cell types for further analysis (Figure 7). These metabolites served as indicators of cellular responses and potential mechanisms of toxicity. The analysis revealed prominent variation in differential metabolite expression profiles among the cell types. In IPEC-J2 cells, all 20 metabolites were significantly downregulated (Figure 7A). Equally, 19 metabolites were significantly downregulated, and 1 was upregulated in PLCs (Figure 7B). PEFs exhibited a mixed response, with 11 metabolites downregulated and 9 upregulated (Figure 7C), while PHs showed downregulation of 16 metabolites and upregulation of 4 (Figure 7D).

**Figure 8:**
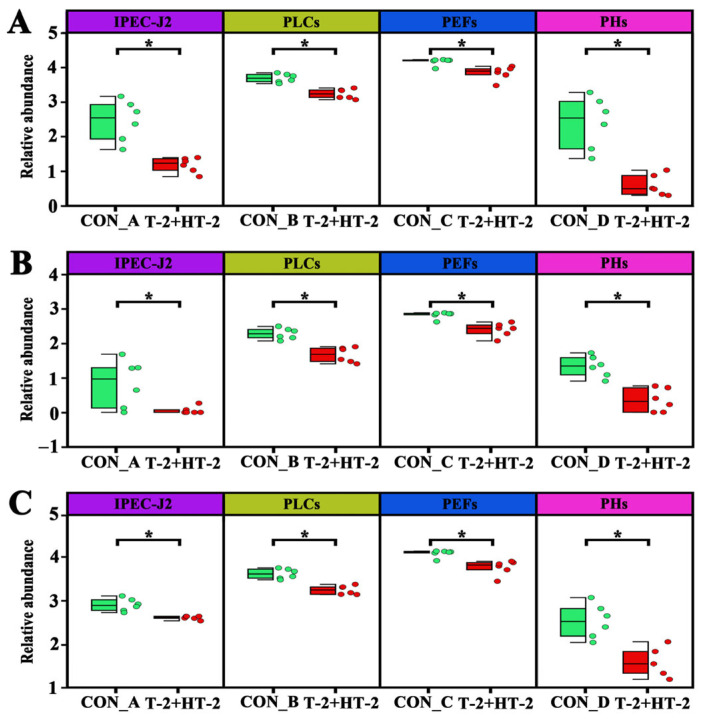
Expression abundance of three representative metabolites in four porcine cell types following co-treatment with T-2 and HT-2 toxins: (**A**) LysoPC (22:6), (**B**) LysoPC (20:5), and (**C**) LysoPC (20:4). In each plot, IPEC-J2 cells are shown in purple, PLCs in yellow, PEFs in blue, and PHs in magenta. Each dot represents an individual sample. Con_A to Con_D represent the toxin-free culture medium controls corresponding to the co-exposure conditions of T-2 and HT-2 in the four different cell lines. In the figure, * denotes a significant difference between groups at *p* < 0.05.

Particularly, three lysophosphatidylcholine species, including LysoPC (22:6), LysoPC (20:5), and LysoPC (20:4) were consistently and significantly downregulated across all four cell types, suggesting a potential shared pathway of disruption under combined exposure of T-2 and HT-2 toxins. The abundance of the three metabolites in the four cell lines is shown in Figure 8, where the three metabolites in the four cells were significantly different compared to the control (*p* < 0.05) with minimal variations observed between replicates.

### 2.8. Metabolic Pathway and Network Analysis

**Figure 9:**
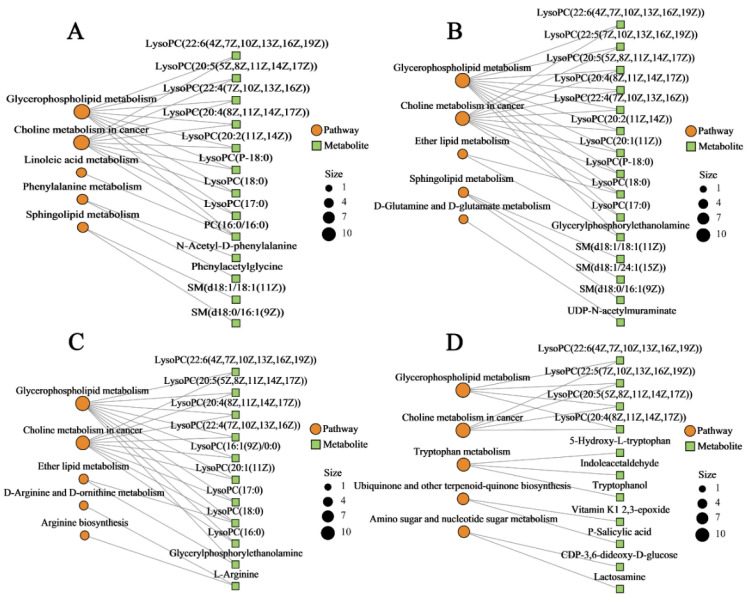
Network diagrams of the relationships between metabolic pathways and associated metabolites in four porcine cells following co-treatment with T-2 and HT-2 toxins: (**A**) IPEC-J2, (**B**) PLCs, (**C**) PEFs, and (**D**) PHs. In each diagram, orange circles represent metabolic pathways, with the circle size proportional to the number of metabolites enriched within the pathway. Green boxes denote the individual enriched metabolites.

KEGG pathway was employed to investigate the enrichment and metabolite network in the four porcine cell types. To construct the metabolic network, the top five enriched pathways were preferred based on their metabolite count. As illustrated in Figure 9, the glycerophospholipid metabolism and choline metabolism in cancer were the most enriched pathways across all four porcine cell types, suggesting that these two pathways could represent common mechanisms of toxin-induced cellular injury. In addition, cellular type-specific enrichment patterns were also observed: phenylalanine metabolism and sphingolipid metabolism were markedly enriched in IPEC-J2 cells (Figure 9A); ether lipid metabolism and sphingolipid metabolism were predominant in PLCs (Figure 9B); ether lipid metabolism was enriched in PEFs (Figure 9C); and ubiquinone and other terpenoid–quinone biosynthesis and tryptophan metabolism were enriched in PHs (Figure 9D).

**Figure 10:**
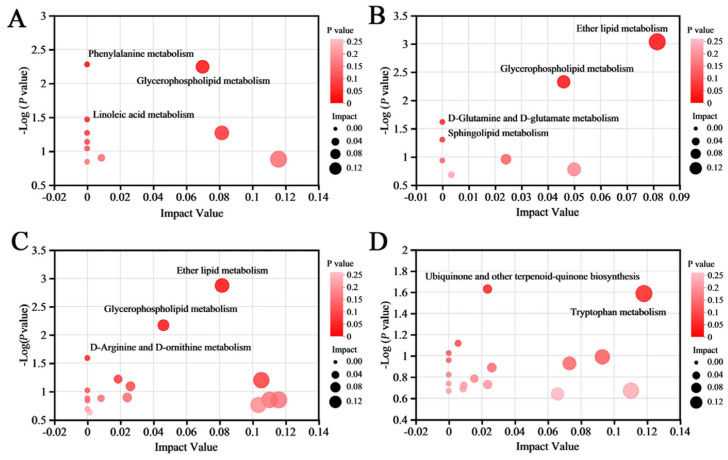
KEGG pathway of differential metabolites in four porcine cell lines following co-treatment with T-2 and HT-2 toxins: (**A**) IPEC-J2, (**B**) PLCs, (**C**) PEFs, and (**D**) PHs. The *x*-axis represents the pathway impact value, reflecting the relative importance of metabolites within each pathway; higher values indicate greater pathway relevance. The *y*-axis denotes the statistical significance of pathway enrichment (−log (*p* value)), with higher values indicating greater enrichment. Each bubble corresponds to a KEGG pathway, with bubble size proportional to its impact score and relevance.

Further pathway topology analysis based on the KEGG database revealed significant alterations in glycerophospholipid metabolism in IPEC-J2, PLCs, and PEFs (*p* < 0.05), with these pathways exhibiting high impact values (Figure 10). In addition, ether lipid metabolism was significantly perturbed in both PLCs and PEFs, displaying the highest impact value among all pathways analyzed in these cell types. In contrast, PHs demonstrated a distinct metabolic response, with tryptophan metabolism being significantly altered, and exhibiting the highest impact value in this cell type.

Overall, comprehensive analysis revealed that disruptions in metabolite expression were strongly linked to alterations in key regulatory pathways. These findings suggest that the observed synergistic toxicity of T-2 and HT-2 toxins is driven by coordinated metabolic dysregulation, which in turn perturbs critical metabolic pathways and ultimately contributes to cellular damage.

## 3. Discussion

This study aimed to investigate the cytotoxic effects of T-2 and HT-2 toxins, both individually and in combination in IPEC-J2, PLCs, PEFs, and PDHs. When exposed to T-2 toxin alone, the relative sensitivity of the cell types followed the following order: IPEC-J2 > PLCs > PEFs > PHs. A similar sensitivity pattern was observed with HT-2 toxin exposure. Notably, co-exposure to T-2 and HT-2 toxins resulted in a synergistic cytotoxic effect at low concentrations, whereas an antagonistic interaction was observed at higher concentrations across the tested cell types.

The IC_50_ values of T-2 toxin in IPEC-J2, PLCs, PEFs, and PHs were determined to be 9.96, 15.83, 17.75, and 43.73 nmol/L, respectively. Previous studies have reported IC_50_ values of T-2 toxin at 24 h as 357 ng/mL for HeLa cells, 63 ng/mL for Bel-7402 cells, and 412 ng/mL for hepatocellular carcinoma cells, indicating cell-type-specific sensitivity to T-2 toxin. Consistent with our findings, a prior study on TM3 Leydig cells reported an IC_50_ between 0.01 and 0.1 μmol/L [26], and another study reported an IC_50_ of 9.3 nmol/L in the porcine intestinal cell line IPEC-1 [27]. In our study, the T-2 toxin exhibited approximately twice the cytotoxicity of HT-2 in three of the four cell types, excluding PEFs. Interestingly, T-2 was 3.2 times more toxic. This difference may be attributed to the primary culture status of these cells, in contrast to the immortalized cell lines used for the other three types. Variations in the cell line origin, culture conditions, exposure duration, and concentration range likely contributed to the observed differences in cytotoxicity [28,29].

The IC_50_ values indicated that T-2 toxin exerted the most pronounced inhibitory effect on the proliferation of IPEC-J2 cells. This finding was in line with previous studies that the concentrations of T-2 and HT-2 toxins in the small intestine of chickens were significantly higher compared to other tissues, supporting the observed sensitivity of porcine intestinal cells in the present study [30]. The cytotoxic response of the four porcine cell types to HT-2 toxin was similarly to that of T-2 toxin, likely due to the shared presence of epoxy sesquiterpene structures in both compounds. Among all tested cell lines, hepatocytes exhibited the lowest sensitivity, which might be attributed to the liver’s intrinsic detoxification capacity [31].

The Chou–Talalay method has been extensively applied to characterize the combinatory effects of binary mycotoxins, including synergistic, additive, and antagonistic interactions [32,33]. In this present study, the CI and DRI value analyses were employed to evaluate the interactive effects of T-2 and HT-2 toxins in four porcine cell lines. Our results demonstrated a dose-dependent interaction: the synergistic effects were observed at low concentrations of T-2 + HT-2 toxins, whereas antagonistic effects predominated at higher doses than that. These findings suggested that co-exposure to T-2 and HT-2 toxins posed a greater toxicological risk than individual exposure at low concentrations but a reduced risk at higher concentrations. Previous studies conducted in our laboratory have a similar synergism at low doses and antagonism at high doses for T-2 and HT-2 combinations, aligning with the current observations [34,35]. Several studies found that T-2 + HT-2 treatment also significantly increased the number of apoptotic cells and the apoptotic ratio [36]. Other mycotoxin studies have shown that OTA and fumonisin B1 presented synergetic cytotoxic effects on rat liver cells (BRL) by inducing apoptosis [37]. In contrast, the admetSAR-predictive model was used to assess the toxicity of T-2 and HT-2 toxins in human hepatocellular carcinoma cells (HepG2), which showed the consistent antagonism across all tested concentrations [14]. This discrepancy stemmed from differences in the biological models employed. These findings indicated that the classification of mycotoxin interactions is model-dependent, with divergent results from different cellular systems in vitro or in vivo [38].

Untargeted metabolomic profiling was conducted to elucidate the synergistic toxicological effects of T-2 and HT-2 toxins in four different porcine cell lines (IPEC-J2, PLCs, PEFs, and PHs). Co-exposure to T-2 and HT-2 resulted in the metabolic perturbations with statistical significance, most notably characterized by the pronounced downregulation of LysoPC (22:6), LysoPC (20:5), and LysoPC (20:4) in all cell types. Since LysoPCs are primarily generated via phospholipase A2 activity, the consistent downregulation of LysoPCs across all cell lines raises the possibility that phospholipase function may be impaired under T-2 and HT-2 co-exposure [39]. Similar studies have demonstrated that LysoPCs represented a prominent class of differentially expressed metabolites associated with renal injury induced by combined exposure to OTA and AFM1 [24]. LysoPCs were bioactive lipid molecules that played the essential roles in reproductive and vascular development and were implicated in multiple pathological processes, including atherosclerosis, inflammation, neurological disorders, and cancer [40,41]. A balanced ratio of LysoPCs to phosphatidylcholines (PCs) was essential for the maintenance of normal physiological functions [42,43]. Therefore, the consistent downregulation of these metabolites indicated that the damage of membrane lipid homeostasis was a common and vulnerable target of the synergistic toxicity caused by T-2 and HT-2 co-exposure in all four porcine cell lines. This disruption of LysoPCs/PCs metabolism could be contributed to an underlying convergent mechanism of toxicity in different porcine cell lines.

Pathway enrichment and topology analyses further supported the changes in these metabolites. Glycerophospholipid metabolism was significantly disrupted in IPEC-J2, PLCs, PEFs, and PHs, and the high impact values and the enrichment of polymetabolites suggested that this pathway played a significant role in mediating cellular injury. Alterations in tryptophan metabolism observed in hepatocytes may reflect impaired hepatic function, as the liver plays a central role in regulating tryptophan catabolism [44]. This pathway was essential for maintaining membrane integrity and facilitating lipid signaling, suggesting that the co-exposure to T-2 and HT-2 mycotoxins led to the disruption of cell membrane structure and function in these cell types [45]. At the cellular level, this observation was consistent with previous studies demonstrating that trichothecene mycotoxins could disrupt membrane lipid metabolism and induce oxidative stress and apoptosis [46]. Another study reported that exposure to another mycotoxin, called aflatoxin B1, induced hepatomegaly and histopathological alterations in goats, accompanied by significant disruptions in glycerophospholipid metabolism and choline metabolism associated with oncogenic pathways [47]. Furthermore, ether lipid metabolism was notably altered in PLCs and PEFs, reinforcing the notion that lipid homeostasis is profoundly affected. These alterations might result from the combined effects of multiple mycotoxins, and the dysregulation of ether lipid biosynthesis has been implicated in the pathogenesis of various diseases, including neurodegenerative disorders, cancer, and metabolic syndromes [48].

Interestingly, PHs exhibited distinct metabolic processes, with tryptophan metabolism as the most significantly altered pathway. Furthermore, the pathway of tryptophan metabolism yielded several neuroactive and immunoregulatory metabolites, which were implicated in neuronal function and stress responses [49]. This observation suggested that PHs displayed a unique sensitivity to T-2/HT-2 co-exposure, which resulted in the cellular damage induced by the alterations of the ether lipid metabolism. Future investigations should assess the reversibility of these metabolic alterations and elucidate the function in vivo, particularly under prolonged exposure.

## 4. Conclusions

In summary, T-2 and HT-2 toxins exhibited dose-dependent cytotoxicity across four porcine cell lines, with IPEC-J2 showing the highest sensitivity. Co-exposure further reduced cell viability, displaying synergistic effects at low doses and antagonistic effects at higher doses. Metabolomic analysis revealed a pronounced and uniform downregulation of LysoPC (22:6), LysoPC (20:5), and LysoPC (20:4) in all cell lines, indicating a common vulnerability in membrane phospholipid composition. Glycerophospholipid metabolism was the most significantly disrupted pathway across all cell types, while ether lipid metabolism was notably perturbed in PLCs and PEFs. PHs uniquely exhibited marked alterations in tryptophan metabolism, indicating cell-type-specific metabolic responses. These findings highlight both common and distinct toxicological mechanisms induced by T-2/HT-2 co-exposure, emphasizing the importance of cell-specific assessments. The integration of cytotoxicity assays with untargeted metabolomics provided novel mechanistic insights and may inform risk assessment frameworks. Future studies involving transcriptomics, proteomics, and in vivo validation are warranted to fully elucidate systemic toxicity and support the development of targeted strategies to mitigate multi-mycotoxin risks in livestock production.

## 5. Materials and Methods

### 5.1. Reagents

PHs were obtained from Qingqi Biotechnology Development Co., Ltd. (Shanghai, China). PLCs were purchased from Shanghai Kelei Biotechnology Co., Ltd. (Shanghai, China), while IPEC-J2 was sourced from Shanghai Baiye Biotechnology Center (Shanghai, China). PEFs were established and preserved at the Shanghai Key Laboratory of Agri-Genetics and Breeding (Shanghai, China).

T-2 toxin and HT-2 toxin powders (purity > 99%) were obtained from Pribolab Pte. Ltd. (Qingdao, China). Sterile stock solutions of T-2 toxin (2 mmol/L) and HT-2 toxin (2 mmol/L) were prepared in dimethyl sulfoxide (DMSO; Sigma Aldrich, Louis, MO, USA) and stored at −20 °C in the dark. Working solutions were diluted in Dulbecco’s Modified Eagle’s Medium (DMEM; Gibco, Waltham, MA, USA) supplemented with 10% fetal bovine serum (FBS; Gibco, Waltham, MA, USA) and 1% antibiotic–antimycotic solution (10,000 U/mL penicillin and 10,000 μg/mL streptomycin; Gibco, Waltham, MA, USA). The final DMSO concentration was kept below 0.1% (*v/v*), a level previously shown to have no adverse effects on cellular parameters. Unless otherwise specified, all additional chemicals were purchased from Sigma-Aldrich (St. Louis, MO, USA).

### 5.2. Cell Culture and Treatments

The four cell lines were cultured in DMEM supplemented with 10% FBS and 1% penicillin–streptomycin solution in a 25 cm^2^ flask, with a humidity-controlled atmosphere of 5% carbon dioxide (CO_2_) at 37 °C. For cytotoxicity assays, an approximately 100 μL cell suspension at a density of 5 × 10^3^ cells/well was seeded into a 96-well plate. After reaching about 80% confluence, the medium was replaced by a DMEM containing various concentrations of T-2, HT-2, and T-2 + HT-2 for 24 h (Table S1). Untreated control wells received only the blank culture medium.

### 5.3. Cell Viability Assay

The enhanced cell-counting Kit-8 (CCK-8) used to perform the cell viability assays was obtained from Shanghai Biosai Biotechnology Co., Ltd. (Shanghai, China). Approximately 100 μL cell suspension with a density of 5 × 10^3^ cells/well was seeded into 96-well plates. After 24 h of incubation, the cells were treated with different doses of T-2, HT-2, and T-2 + HT-2 toxins for an additional 24 h. Changes in cell growth and morphology were monitored during the exposure period. Following treatment, 10 μL of CCK-8 solution was added into each well, and the plates were incubated at 37 °C for 2 h. The absorbance at 450 nm was measured using a microplate reader (Thermo Fisher Scientific, Waltham, MA, USA), and obtained values were normalized to the untreated control cells. Wells containing medium without cells were set up as blank control. At least six replicates were considered for each experimental group.

The cell viability (%) was calculated as follows:

$$P=\frac{{OD}_{2}-{OD}_{0}}{{OD}_{1}-{OD}_{0}}\times 100\%$$

where *P* was the survival rate, *OD*_0_ was the absorbance of the blank group (medium only), *OD*_1_ represented the absorbance of the untreated control group, and *OD*_2_ represented the absorbance of the experimental (treated) group.

### 5.4. Combined Index Analysis of Mycotoxin Mixtures

The dose–effect relationships for T-2, HT-2, and their combination (T-2 + HT-2) were calculated using the median-effect equation proposed by Chou and Talalay [50]. The combination index (CI) values for T-2 + HT-2 were described in our previous work and calculated using Compusyn 1.0 software to analyze the interactive effect [16], as shown in Table S2. Furthermore, the dose reduction index (DRI) can be used to evaluate the synergistic effect. The DRI indicates how many times the dose of each toxin can be reduced in the combination treatment to achieve a given level of effect (e.g., x% inhibition) compared to the dose required when each toxin is used alone to achieve the same effect. A higher DRI suggests a stronger synergistic interaction [51].

### 5.5. Extraction of Cell Metabolites

Four types of porcine cells were digested and seeded into 6-well plates at a density of 1 × 10^5^ cells/mL, with 2 mL serum-free DMEM added to each well. The concentrations of T-2, HT-2, and their combination (T-2 + HT-2) used for co-exposure were selected based on the doses that reduced cell viability to approximately 25%. Detailed toxin concentrations and experimental groups are provided in Table S3. Each experimental group included at least six biological replicates, and corresponding control groups were maintained in a toxin-free culture medium.

Metabolite extraction and analytical procedures were adapted with minor modifications from previously established protocols [52]. Briefly, after 24 h of exposure, the culture supernatant was collected by centrifugation at 2000 rpm/min. A 400 μL aliquot of the supernatant was mixed with 1200 µL of pure methanol, vortexed for 1 min, and incubated in an ice bath for 5 min. The mixture was then centrifuged at 13,000 r/min for 10 min at 4 °C. The supernatant was freeze-dried using a SCIENTZ-10N freeze dryer (Ningbo, China). Lyophilized samples were reconstituted in 60 μL of 1% formic acid aqueous solution, and the final supernatant was filtered through a 0.22 μm membrane prior to analysis by liquid chromatography–quadrupole time-of-flight mass spectrometry (LC-Q-TOF/MS; Waters, Milford, MA, USA). Additionally, 10 μL from each replicate were pooled, mixed, and used as a quality control (QC) sample.

### 5.6. LC-Q-TOF/MS Conditions

An LC-Q-TOF/MS system was used for the metabolomic analysis. Chromatographic separation was performed on an XBridge BEH C18 column (100 mm × 3.0 mm, 2.5 μm; Waters, Milford, MA, USA) at a column temperature of 40 °C. The injector was maintained at room temperature, with an injection volume of 5 μL and a flow rate of 0.4 mL/min. The mobile phase consisted of 0.1% formic acid in water (A) and acetonitrile (B), with a gradient elution program detailed in Table S4. Mass spectrometric analysis was conducted using an electrospray ionization in positive and negative ion modes (ESI+/−). The specific mass spectrometry parameters were provided in Table S5.

### 5.7. Statistical Analysis and Data Visualization

Cytotoxicity parameters and their CI and DRI values were analyzed using version 3.0.1 computer software (ComboSyn, Inc., Paramus, NJ, USA) to assess the dosage–effect curve for individual and combined mycotoxins. All statistical analyses were performed using one-way of variance (ANOVA) followed by the least significant difference (LSD) post hoc test to determine statistical significance using SPSS 26.0 for Windows (SPSS Inc., Chicago, IL, USA). Each experimental value represented the mean ± standard deviation (SD) of at least six independent replicates. When the *p* value is <0.01, the statistical difference was considered significant. Metabolomics data were processed using Progenesis QI v2.1 software (Waters Corporation, Milford, MA, USA) for alignment, peak extraction, sample grouping, and deconvolution. Multivariate analyses were performed using the Majorbio online analysis platform (https://www.majorbio.com/ (accessed on 23 March 2025)). Significantly altered metabolites were identified using the following criteria: variable importance in projection (VIP) > 1, fold change (FC) > 1 or <1, and *p* < 0.05 [53]. A pathway enrichment analysis of these metabolites was conducted using the Kyoto Encyclopedia of Genes and Genomes (KEGG).

## Figures and Tables

**Table 1:** IC_50_ values for T-2 and HT-2 in four different porcine cell lines.

Cell Lines	T-2 (nmol/L)	HT-2 (nmol/L)	Ratio of HT-2 to T-2
IPEC-J2	9.96 ± 0.51 ^c^	20.55 ± 0.13 ^b^	2.06
PLCs	15.83 ± 1.16 ^b^	34.69 ± 2.38 ^b^	2.19
PEFs	17.75 ± 1.59 ^b^	57.80 ± 1.41 ^a^	3.26
PHs	43.72 ± 2.21 ^a^	98.92 ± 6.06 ^a^	2.26

The cytotoxic effects of T-2 and HT-2 were evaluated in four porcine cell lines using at least six serial dilutions of each toxin. IC_50_ values for individual toxins, as well as their combination ratios, were determined using CompuSyn 1.0 software. Results are presented as mean ± SD. Different lowercase superscript letters (e.g., a, b, c) denoted statistically significant differences between each group (*p* < 0.05), with the absence of shared letters indicating significant variation.

**Table 2:** Types of interactions observed following exposure to binary combinations of T-2 and HT-2 toxins in four porcine cell lines.

Cells Lines	Toxins	Ratio	25% Cytotoxicity			50% Cytotoxicity			75% Cytotoxicity		
			CI Values		DRI	CI Values		DRI	CI Values		DRI
IPEC-J2	T-2	1:2	0.88	+	2.75	1.00	±	1.98	1.16	–	1.43
	HT-2				1.94			2.05			2.16
PLCs	T-2	1:2	0.43	+ + +	5.30	1.65	– – –	1.23	6.38	– – – –	0.28
	HT-2				4.10			1.20			0.35
PEFs	T-2	1:3	0.77	+ +	2.27	1.09	±	1.76	1.57	– – –	1.37
	HT-2				3.05			1.91			1.20
PHs	T-2	1:2	0.10	+ + + +	18.61	1.04	±	1.82	10.69	– – – – –	0.18
	HT-2				21.02			2.04			0.20

**Table 3:** Details of seven metabolites with clear biological function or pathway annotations.

HMDB ID	Metabolite	Chemical Formula	*m*/*z*
HMDB0010404	LysoPC(22:6)	C30H50NO7P	612.33
HMDB0010397	LysoPC(20:5)	C28H48NO7P	586.32
HMDB0010396	LysoPC(20:4)	C28H50NO7P	588.33
HMDB0010393	LysoPC(20:3)	C28H52NO7P	590.35
HMDB0010386	LysoPC(18:2)	C26H50NO7P	564.33
HMDB0011491	LysoPE(0:0/22:1)	C27H54NO7P	580.37
HMDB0035264	Avenestergenin A1	C38H55NO7	618.38

## Data Availability

The original contributions presented in this study are included in the article/Supplementary Materials. Further inquiries can be directed to the corresponding author(s).

## Supplementary Materials

The following supporting information can be downloaded at https://www.mdpi.com/article/10.3390/toxins17080381/s1: Table S1. Mycotoxins concentrations used in the current study. Table S2. Classification criteria for the combined toxic effects and the corresponding symbols. Table S3. Detailed experimental groupings and specific toxin concentrations established for co-toxicity assessments in metabolomics analyses. Table S4. Gradient elution program. Table S5. Mass spectral parameters in positive and negative ion modes. Figure S1. Quality Control (QC) Sample Evaluation Figure.
