# Supplementary material for: Integrated Cytotoxicity and Metabolomics Analysis Reveals Cell-Type-Specific Responses to Co-Exposure of T-2 and HT-2 Toxins
Source: Toxins (Basel). 2025 Jul 30;17(8):381. doi: 10.3390/toxins17080381 (PMC12389818; doi:10.3390/toxins17080381)
Supplement: Supplementary file 1 [file toxins-17-00381-s001.zip › toxins-3738072-supplementary.pdf]

Integrated Cytotoxicity and Metabolomics Analysis Reveals Cell-Type-Specific Responses to Co-Exposure of T-2 and HT-2 Toxins

Weihua He [1, †], Zuoyin Zhu [2, †], Jingru Xu [2], Chengbao Huang [2], Jianhua Wang [2], Qinggong Wang [1], Xiaohu Zhai [1, *] and Junhua Yang [2, *]

1 Institute of Pet Science and Technology, Jiangsu Agri-Animal Husbandry Vocational College, Taizhou 225300, China;

2 Institute for Agro-Food Standards and Testing Technology, Shanghai Academy of Agricultural Sciences, Shanghai 201403, China.

* Correspondence: zhaixiaohu@jsahvc.edu.cn (X.Z.); yangjunhua303@126.com (J.Y.)

† These authors contributed equally to this work.

Table S1 Mycotoxins concentrations used in the current study.

Cells Lines	T-2 (nmol/L)	HT-2 (nmol/L)
	1.5625	3.125
	3.125	6.25
IPEC-J2	6.25	12.5
PLCs	12.5	25
	25	50
	50	100
	3.125	6.25
	6.25	12.5
PEFs	12.5	25
PHs	25	50
	50	100
	100	200

Abbreviations. IPEC-J2: porcine intestinal epithelial cells, PLCs: porcine Leydig cells, PEFs: porcine ear fibroblasts, PHs: porcine hepatocytes, T-2: T-2 toxins, and HT-2: HT-2 toxins.

Table S2 Classification criteria for the combined toxic effects and the corresponding symbols.

Combination Index (CI)	Types of interactions	Function symbols
<0.10	Extremely strong synergy	+++++
0.10-0.30	Strong synergy	++++
0.30-0.70	General synergy	+++
0.70-0.85	Weak synergy	++
0.85-0.90	Extremely weak synergy	+
0.90-1.10	Approximate summation	±
1.10-1.20	Extremely weak antagonism	−
1.20-1.45	Weak antagonism	−−
1.45-3.30	General antagonism	−−−
3.30-10.00	Strong antagonism	−−−−
>10.00	Extremely antagonism	−−−−−

Table S3 Detailed experimental groupings and specific toxin concentrations established for co-toxicity assessments in metabolomics analyses.

Concentration (nmol/L)		IPEC-J2	PLCs	PEFs	PHs
T-2+HT-2	T-2	1.5625	3.125	1.5625	3.125
	HT-2	3.1225	6.25	4.6875	6.25
Control	CON_A/B/C/D	0 (CON_A)	0 (CON_B)	0 (CON_C)	0 (CON_D)

The concentrations of T-2 and HT-2 toxins were established for each porcine-derived cell line as follows: 1.5625 nmol/L T-2 and 3.125 nmol/L HT-2 for IPEC-J2 cells; 3.125 nmol/L T-2 and 6.25 nmol/L HT-2 for PLC cells; 1.5625 nmol/L T-2 and 4.6875 nmol/L HT-2 for PEF cells; and 3.125 nmol/L T-2 and 6.25 nmol/L HT-2 for PH cells. Corresponding control groups were maintained in toxin-free medium under identical culture conditions for 24 hours: Control-A (CON_A) for IPEC-J2, Control-B (CON_B) for PLC, Control-C (CON_C) for PEF, and Control-D (CON_D) for PH cells.

Table S4 Gradient elution program.

Time/min	A%	B%
0	95	5
1	95	5
12	0	100
16	0	100
16.1	95	5
18	95	5

Table S5 Mass spectral parameters in positive and negative ion modes.

Parameter	Positive ion	Negative ion
Spray Voltage (V)	3800	3000
Capillary Temperature (°C)	320	320
Aux gas heater temperature (°C)	350	350
Sheath Gas Flow Rate (Arb)	35	35
Aux gas flow rate (Arb)	8	8
S-lens RF level	50	50
Mass range (m/z)	70-1000	70-1000
Full ms resolution	70000	70000
MS/MS resolution	17500	17500
NCE/stepped NCE	20, 40	20, 40

Figure S1 Quality Control (QC) Sample Evaluation Figure.

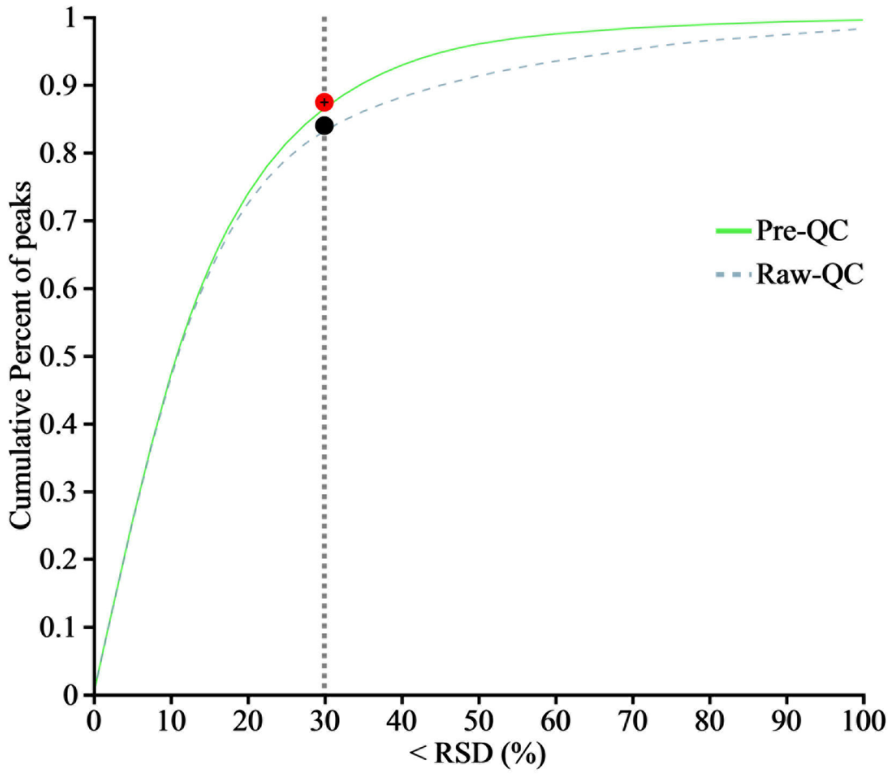

The x-axis represents the RSD (%) value, i.e., the standard deviation divided by the mean, while the y-axis indicates the cumulative proportion of ion peaks. For the overall dataset, if RSD < 30% and the cumulative proportion of peaks > 0.7, the data are considered qualified and stable. The dashed line represents the sample data before preprocessing, and the solid line represents the sample data after preprocessing.
